# Anorexia nervosa polygenic risk, beyond diagnoses: relationship with adolescent disordered eating and behaviors in an Australian female twin population

**DOI:** 10.1017/S0033291724001727

**Published:** 2024-10

**Authors:** Madeleine Curtis, Lucia Colodro-Conde, Sarah E. Medland, Scott Gordon, Nicholas G. Martin, Tracey D. Wade, Sarah Cohen-Woods

**Affiliations:** 1 Discipline of Psychology, College of Education, Psychology, and Social Work, Flinders University, Adelaide, SA, Australia; 2 Blackbird Initiative, Flinders Institute for Mental Health and Wellbeing, Flinders University, Adelaide, SA, Australia; 3 QIMR Berghofer Medical Research Institute, Brisbane, Australia; 4 Flinders Centre for Innovation in Cancer, College of Medicine and Public Health, Flinders University, Adelaide, SA, Australia

**Keywords:** anorexia nervosa, disordered eating, eating disorder, genetics, polygenic risk

## Abstract

**Background:**

It is well established that there is a substantial genetic component to eating disorders (EDs). Polygenic risk scores (PRSs) can be used to quantify cumulative genetic risk for a trait at an individual level. Recent studies suggest PRSs for anorexia nervosa (AN) may also predict risk for other disordered eating behaviors, but no study has examined if PRS for AN can predict disordered eating as a global continuous measure. This study aimed to investigate whether PRS for AN predicted overall levels of disordered eating, or specific lifetime disordered eating behaviors, in an Australian adolescent female population.

**Methods:**

PRSs were calculated based on summary statistics from the largest Psychiatric Genomics Consortium AN genome-wide association study to date. Analyses were performed using genome-wide complex trait analysis to test the associations between AN PRS and disordered eating global scores, avoidance of eating, objective bulimic episodes, self-induced vomiting, and driven exercise in a sample of Australian adolescent female twins recruited from the Australian Twin Registry (*N* = 383).

**Results:**

After applying the false-discovery rate correction, the AN PRS was significantly associated with all disordered eating outcomes.

**Conclusions:**

Findings suggest shared genetic etiology across disordered eating presentations and provide insight into the utility of AN PRS for predicting disordered eating behaviors in the general population. In the future, PRSs for EDs may have clinical utility in early disordered eating risk identification, prevention, and intervention.

Disordered eating encompasses all DSM-5 eating disorders (EDs), as well as disordered eating-related behaviors and cognitions that may not meet diagnostic criteria but still cause significant distress and impairment (American Psychiatric Association [APA], 2013). This includes cognitions such as an intense fear of weight gain or a distortion in body image, and behaviors such as food restriction, binge-eating, or self-induced vomiting (APA, 2013). Both behavioral and cognitive disordered eating symptoms can both cause significant physical and psychological impairment, regardless of whether criteria for a specified clinical ED diagnosis are met (APA, 2013; Wilkop, Wade, Keegan, & Cohen-Woods, 2023).

Disordered eating typically develops during adolescence and disproportionately affects females, with a recent study reporting point prevalence of any ED among Australian adolescents of 32.9% for females and 12.8% for males (Mitchison et al., 2020). ED presentations are dynamic and change with age and in duration. High rates of diagnostic crossover at 12-month (Forbush et al., 2018) and 30-month follow-up periods have been reported (Milos, Spindler, Schnyder, & Fairburn, 2005), with restrictive EDs first to emerge in early adolescence and binge/purge symptoms developing later in adolescence or early adulthood (Fairburn, Cooper, & Shafran, 2003; Hudson, Hiripi, Pope, & Kessler, 2007). Disordered eating symptoms often exist prior to the development of a clinical ED with symptoms worsening across childhood, and through adolescence to adulthood (McClelland, Robinson, Potterton, Mountford, & Schmidt, 2020). There is a need for research into the broader construct of disordered eating, which is not restricted to specific diagnostic criteria, and to capture measurements across adolescence – a dynamic period of growth and change.

Many risk factors have been identified, with genetic influences now well-established in disordered eating and across clinical EDs (Fairweather-Schmidt & Wade, 2014). Psychiatric disorders are polygenic with thousands of genetic variants contributing to risk for all disorders examined to date, each with a very small, additive effect (Visscher, Yengo, Cox, & Wray, 2021). Genome-wide association studies (GWASs) are leveraged to identify genetic risk factors for polygenic traits and involve testing hundreds of thousands of genetic variants across the genome (Hübel, Leppä, Breen, & Bulik, 2018). Anorexia nervosa (AN) is the only clinical ED to have been the subject of large meta-analytic GWASs, with large-scale recruitment for GWASs on bulimia nervosa (BN) and binge eating disorder (BED) currently underway (Bulik et al., 2022; Steiger & Booij, 2020). A recent GWAS on BED has been published; however this is relatively small and case status was predicted using machine learning to distinguish between clinically diagnosed BED and obesity (Burstein et al., 2023).

The largest AN GWAS to date by Watson et al. (2019) identified eight genome-wide significant loci associated and estimated 11–17% of phenotypic variation could be attributed to single-nucleotide polymorphism variation captured. The polygenic risk score (PRS) generated from this GWAS, however, accounted for just 1.7% of phenotypic variation (Watson et al., 2019). This means there are still many more risk variants for AN to identify, which will likely happen as sample sizes increase and statistical power improves (Bulik et al., 2022). With no differences in genetic variation between those with and without binge eating AN subtypes (Watson et al., 2019), and twin studies showing significant overlap in genetic risk factors between lifetime AN, BN or BED, and other specified feeding or eating disorders (OSFED) (Fairweather-Schmidt & Wade, 2014), it is clearly worthwhile to investigate if other eating disorders – and disordered eating behaviors – are also associated with AN polygenic risk, and if these relationships are observed also at the population level.

Few studies have investigated whether AN PRSs predict other specific disordered eating behaviors with mixed findings and restricted to limited cohorts. Within the Adolescent Brain Cognitive Development Study (ABCD) cohort no relationship between AN PRS and ED psychopathology at ages 9–11 was reported, based on a lifetime ED screener (Westwater et al., 2023). Similarly, Abdulkadir et al. (2022) reported no association between AN PRS and presence or absence of ED symptoms based on the Youth Risk Behavior Surveillance System surveys at ages 14, 16, and 18 in the Avon Longitudinal Study of Parents and Children (ALSPAC) cohort, or surveys capturing fear of weight gain, thin ideal internalization, weight and shape concern, or body dissatisfaction individually. There was no investigation of global disordered eating across these symptom scales. Yilmaz et al. (2023) also investigated the ALSPAC cohort but with a slightly larger sample and sex-specific analyses for the first time. Here they identified a relationship between AN PRS and eating disorder not otherwise specified (EDNOS)/purging disorder, presence of any ED, and compulsive exercise at age 14 – in girls specifically. All studies categorized individuals with dichotomous yes/no responses in relation to disordered eating behaviors and relied on parent report (except for ALSPAC age 16 and 18 measures). Where scales were used they focused on one cognitive feature of EDs (such as body dissatisfaction or weight and shape concern) with no global capture of disordered eating as a global construct across cognitions.

To date no study has investigated the relationship between AN PRS and disordered eating more broadly as a phenotype within the population, using global measures of eating psychopathology and not just measures of individual aspects of symptomatology or behaviors. The high levels of impairment experienced by those with disordered eating, even when not meeting diagnostic criteria for a specified ED, emphasizes the need for research on the broader phenotype of disordered eating (Wilkop et al., 2023). With ED presentations changing across ages while genetic variants are stable from birth (Brookes, 1999; Fairburn et al., 2003; Forbush et al., 2018), it is logical to ascertain if genetic variants associated with AN are also associated with disordered eating broadly. If PRSs can quantify genomic risk for disordered eating at an individual level, and is later determined to predict conversion from non-clinical disordered eating to clinical disorder, PRSs may have clinical utility to assist with targeted early intervention and prevention efforts in the future.

This study aimed to investigate whether PRSs for AN could predict disordered eating in an Australian female twin population. To explore this we tested whether AN PRS predicted overall levels of disordered eating and if a higher AN PRS was associated with any lifetime disordered eating behaviors, specifically avoidance of eating, objective bulimic episodes, self-induced vomiting, or driven exercise.

## Method

### Participants and procedure

**Figure 1.**
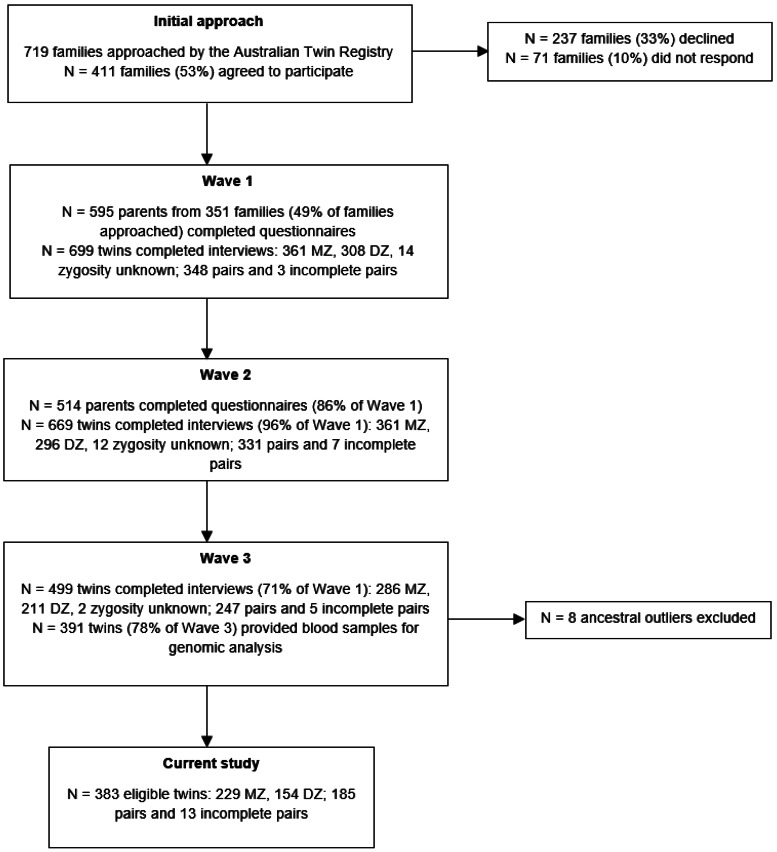
Flow diagram of data collection.

The target cohort for the present study was obtained from a longitudinal study of Australian adolescent female twins. The data collection process has been described in detail in several published studies (Fairweather-Schmidt & Wade, 2014, 2017; Wade, Byrne, & Bryant-Waugh, 2008; Wade et al., 2013; Wade & O'Shea, 2015; Wilksch & Wade, 2009). Data were collected longitudinally over three time periods (wave 1, wave 2, wave 3; see Fig. 1). There was a mean duration of 1.5 years (s.d. = 0.17) between waves 1 and 2, and 2.96 years (s.d. = 0.27) between waves 2 and 3. Wave 1 included 669 participants (mean age 13.96, s.d. = 0.80), wave 2 included 669 (mean age 15.1, s.d. = 0.83), and wave 3 included 499 participants (mean age 16.9, s.d. = 0.70). Participants who were retained at wave 3 did not differ significantly from those who were not retained with respect to age (*p* = 0.709), body mass index (BMI) centile (*p* = 0.704), or disordered eating global scores (*p* = 0.266). Response rates are detailed in Fig. 1 and comparable with other large Australian epidemiological twin studies with multiple data collection points (Stice, Marti, & Rohde, 2013; Wade, Bergin, Tiggemann, Bulik, & Fairburn, 2006). Waves 1 and 2 consisted of a telephone-administered interview with the twins, and a self-report questionnaire sent to parents of the twins. Wave 3 consisted of the telephone interview only. Blood samples were collected from twins at wave 3 (*n* = 391) using ethylenediaminetetraacetic acid (EDTA) collection tubes for genomic analysis. Ethical approval was provided by the Flinders University Clinical Research Ethics Committee (no. 115/07) and written informed consent was obtained from all participants.

Participants included in the present study had genomic data available and completed the telephone interview at each wave (*n* = 391). Eight participants were identified as ancestral outliers and excluded from the present study leaving a total sample of 383 participants. Participant characteristics are displayed in Table 1. The included sample consisted of 185 twin pairs and 13 incomplete twin pairs, of which 229 participants were monozygotic twins (*n* = 110 complete pairs) and 154 were dizygotic twins (*n* = 75 complete pairs). Mean age of the included participants was 14.01 (s.d. = 0.78) at wave 1, 15.15 (s.d. = 0.82) at wave 2, and 16.95 (s.d. = 0.83) at wave 3, with an overall range of 12.74–19.84 years. Participants were of European ancestry and had an average socioeconomic indexes for areas of 100.95 (s.d. = 10.80).

**Table 1.** Participant characteristics

	Wave 1 (*n* = 383)	Wave 2 (*n* = 383)	Wave 3 (*n* = 383)
Age			
Mean (s.d.)	14.01 (0.78)	15.15 (0.82)	16.95 (0.83)
Range	12.74–15.88	13.82–16.97	15.59–19.84
BMI centile			
Mean (s.d.)	53.8 (30.25)	55.20 (28.96)	51.57 (28.71)
Range	0–99.91	0–99.89	0–99.98
Global EDE score			
Mean (s.d.)	0.44 (0.65)	0.45 (0.74)	0.42 (0.69)
Range	0–4.02	0–4.83	0–4.71

### Measures

As this cohort study commenced prior to 2008, disordered eating outcomes were assessed using the 12th edition of the Eating Disorder Examination (EDE) (Fairburn, Cooper, & O'Connor, 1993) slightly modified for use with children, in line with previous recommendations (Bryant-Waugh, Cooper, Taylor, & Lask, 1996; Wade et al., 2008). The EDE consists of 22 items measured on a 7-point Likert scale (scores ranging 0–6) and assesses range, severity, and frequency of disordered eating symptoms over the previous 28 days. Items cover four subscales including eating concern (e.g. preoccupation with food, fear of losing control over eating), weight concern (e.g. dissatisfaction with weight, fear of weight gain), shape concern (e.g. dissatisfaction with body shape, fear of becoming fat), and dietary restraint (e.g. attempts to restrict food intake, avoidance of certain foods). The EDE also includes diagnostic questions addressing behaviors over the previous 3 months. In the present sample, the EDE was revised with addition of lifetime diagnostic questions, including the age range in which the behavior occurred.

The EDE was administered to participants as a semi-structured interview via telephone at each wave of data collection. Interviews were conducted by postgraduate clinical psychology trainees (*N* = 16) who were trained in the use of the EDE. Twins were interviewed at separate times and with a different interviewer for each twin in the family. Independent ratings of 20 randomly selected interviews showed high interrater reliability for each of the four EDE subscales (Wade et al., 2008).

#### Global measure of disordered eating

Participants' global scores on the EDE were used as a continuous measure of disordered eating. Global scores were generated by calculating the mean item score across the eating concern, weight concern, shape concern, and dietary restraint subscales. Scores range from 0 to 6, with higher scores indicating greater symptom severity. To quantify the most pronounced eating symptoms demonstrated by each participant during the study, we used the highest global EDE score recorded across all three waves of data collection as our continuous measure of disordered eating. Global EDE scores are a useful and widely utilized measure of eating pathology and studies report global scores to be a stronger indicator of overall eating pathology than individual subscale scores (Friborg, Reas, Rosenvinge, & Rø, 2013; Jenkins & Rienecke, 2022). Global EDE scores also accurately discriminate between those with and without a clinically diagnosed ED (Aardoom, Dingemans, Slof Op't Landt, & Van Furth, 2012; Mond et al., 2008). In our sample construct validity was indicated with global scores significantly higher among those meeting diagnostic thresholds for key disordered eating symptoms, and convergent validity was demonstrated with medium-large correlations with Eating Disorder Inventory (Garner, Olmstead, & Polivy, 1983) measures of interoceptive awareness and drive for thinness (Fairweather-Schmidt & Wade, 2015; Wade et al., 2008). Global scores also demonstrated excellent internal reliability in our sample (Cronbach's *α* = 0.93 at waves 1 and 3) (Fairweather-Schmidt & Wade, 2015).

#### Lifetime disordered eating behaviors

The EDE telephone interview also included several behavioral frequency questions, which have demonstrated high interrater, and test–retest, reliability across studies (Berg, Peterson, Frazier, & Crow, 2012). Behavioral frequency questions addressed a 3-month period and assessed the presence of both current and lifetime disordered eating behaviors. As we were interested in disordered eating behaviors at any point throughout the participants' life, we used the lifetime diagnostic questions. Four lifetime behaviors were investigated: avoidance of eating, objective bulimic episodes, self-induced vomiting, and driven exercise. Criteria for the presence (yes/no) of each lifetime behavior are outlined in Table 2.

**Table 2.** Lifetime behaviors assessed in the telephone interview

Behavior	Criteria to meet threshold
Avoidance of eating	Person has gone for periods of 8 or more waking hours without eating anything in order to influence shape or weight on more than half the days each week for a 3-month period.
Objective bulimic episodes	Behavior occurred at least twice a week for a 3-month period, with breaks of no more than 2 weeks.
Self-induced vomiting	Behavior occurred at least twice a week for a 3-month period, with breaks of no more than 2 weeks.
Driven exercise	Driven or compulsive exercise occurred for at least 1 h, 5 days a week for a 3-month period, with breaks of no more than 2 weeks.^a^

a Only exercise for weight or shape reasons was included (questions were asked separately for competitive sport and other forms of exercise).

### PRS calculation

DNA was extracted from whole blood of 383 participants and genotyped using the Infinium Global Screening Array V.1 (Illumina, CA) at Erasmus Medical Centre (Rotterdam). Standard quality control procedures were applied and imputation was performed using the Michigan Imputation Server (see online Supplementary materials). AN PRSs were calculated in PLINK version 1.9 (Chang et al., 2015) using GWAS summary statistics from the Eating Disorders Working Group of the Psychiatric Genomics Consortium (PCG-ED) Freeze 2 AN sample (Watson et al., 2019) (see online Supplementary materials). Australian and New Zealander participants were excluded to avoid potential crossover between base and target datasets. To optimize prediction accuracy and identify the *p* value threshold with the greatest prediction for each disordered eating outcome, PRSs were calculated for eight different *p* value thresholds.

### Statistical analyses

Genome-wide complex trait analysis (GCTA) genome-based restricted maximum-likelihood analyses (Yang, Lee, Goddard, & Visscher, 2011) were used to test association between AN PRS and global disordered eating scores, and presence of lifetime disordered eating behaviors, at eight *p* value thresholds, controlling for covariates. We controlled for relatedness using GCTA to calculate the genetic relationship matrix for our target sample. The first five genetic principal components were included as covariates to control for population stratification (Price et al., 2006). Additional covariates included were participant age and BMI centile, reported at the same data collection time point as their highest global EDE score. Age was included to control for age-related differences in disordered eating (Fairburn et al., 2003; McClelland et al., 2020) and BMI has been associated with disordered eating behaviors and attitudes (Goldschmidt, Aspen, Sinton, Tanofsky-Kraff, & Wilfley, 2008). We used BMI centile (BMI-for-age) in place of BMI as it is considered more accurate than BMI for children (Centre for Disease Control and Prevention [CDC], 2022). We corrected for multiple testing by calculating the false-discovery rate (FDR)-adjusted *p* values (*Q*) using the Benjamini and Hochberg method (Benjamini & Hochberg, 1995). We report results as proportion of variance in the dependent variable (disordered eating) explained by the independent variable (PRS) (*r*^2^).

## Results

### Disordered eating global score

**Figure 2.**
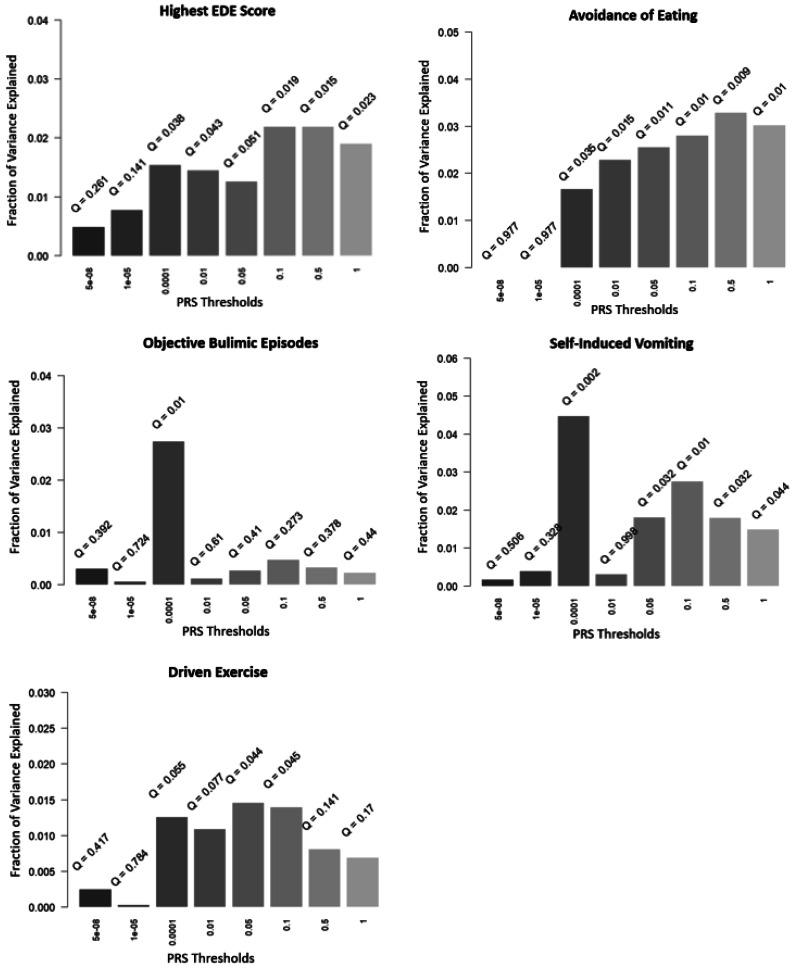
Associations between AN PRS and disordered eating outcomes at eight *p* value thresholds.

Participants had a mean highest global EDE score of 0.73 (s.d. = 0.90, range 0–4.83). The highest global EDE scores were reported by 36% of participants (*n* = 138) at wave 1, 30% (*n* = 117) at wave 2, and 33% (*n* = 128) at wave 3. A total of 11% of participants (*n* = 43) scored above what is considered a ‘normal’ EDE-Q score based on Australian community norms (i.e. <1.81) (Mond, Hay, Rodgers, Owen, & Beumont, 2004). The results of all PRS association analyses are presented in Fig. 2. After applying the FDR correction, the AN PRS significantly predicted global disordered eating in the target sample at five of the eight *p* value thresholds, with the most significant association at the *p* < 0.5 threshold (*r*^2^ = 2.19%, *Q* = 0.015).

#### EDE subscales

To explore whether the association between AN PRS and global EDE scores was driven by specific EDE subscales, we repeated the analyses for each EDE subscale individually (online Supplementary materials, Fig. 3). After applying the FDR correction, the AN PRS significantly predicted scores on the weight concern (*r*^2^ = 1.88%, *Q* = 0.031), shape concern (*r*^2^ = 1.95%, *Q* = 0.030), and eating concern (*r*^2^ = 2.17%, *Q* = 0.030) subscales. The most predictive *p* value thresholds were *p* < 0.001 for weight concern, and *p* < 0.5 for both shape and eating concerns. The AN PRS also explained 1.38% of variance in scores on the dietary restraint subscale, however this result was not statistically significant after FDR correction (*Q* = 0.052).

### Lifetime disordered eating behaviors

The lifetime prevalence in our target sample for avoidance of eating, objective bulimic episodes, self-induced vomiting, and excessive exercise were 5%, 4.2%, 1.8%, and 7.8% respectively. Avoidance of eating was significantly associated with the AN PRS at six *p* value thresholds, with *p* < 0.5 generating the most significant association (*r*^2^ = 3.29%, *Q* = 0.009). The AN PRS significantly predicted both self-induced vomiting (*r*^2^ = 4.48%, *Q* = 0.002) and objective bulimic episodes (*r*^2^ = 2.74%, *Q* = 0.010) at *p* < 0.001. Excessive exercise was also significantly associated with the AN PRS after applying the FDR correction at both *p* < 0.05 (*r*^2^ = 1.46%, *Q* = 0.044) and *p* < 0.1 (*r*^2^ = 1.40%, *Q* = 0.045).

## Discussion

This study was the first to comprehensively explore whether AN PRS predicts disordered eating as a broad phenotype in the general population. This was achieved by investigating both a continuous measure of disordered eating symptomology, as well as four specific lifetime disordered eating behaviors. This enabled us to examine whether any association between AN PRS and disordered eating was specific to restrictive behaviors (EDE global scores, avoidance of eating) or extended to disordered eating behaviors of relevance across different EDs (objective bulimic episodes, self-induced vomiting, driven exercise). After applying the FDR correction, the AN PRS was associated with all disordered eating outcome measures, explaining a significant amount of phenotypic variance in global EDE scores (2.19%), avoidance of eating (3.29%), objective bulimic episodes (2.74%), self-induced vomiting (4.48%), and driven exercise (1.46%). As the EDE consists of four subscales measuring different components of disordered eating symptomology, we additionally explored whether the association between AN PRS and EDE global scores was driven by specific subscales. The AN PRS significantly predicted scores on the weight concern, shape concern, and eating concern subscales, but was just short of significance following multiple testing corrections on the dietary restraint subscale. Given restraint is a key feature of AN this is an interesting finding and suggests that the AN PRS may be less predictive of dietary restraint behavior – possibly because dieting can be relatively common – but more predictive of other cognitive disordered eating symptoms such as weight and shape concern. EDE subscales have been widely investigated in the context of both EDs and disordered eating outside of genomics, however it is important to highlight that the validity of the individual EDE subscale scores has been questioned (Wade et al., 2008). The factor structure, as purported by the subscales, is not stable across studies, and so our findings with specific subscales must be couched within this caution. Our findings do however support a relationship between AN PRS and cognitive indicators of disordered eating – not just behavioral.

Our finding that the AN PRS predicted global disordered eating scores in our sample supports the possibility of a shared genetic basis across different ED presentations. Given that this is the first study to apply the AN PRS to a global disordered eating measure, this is a novel finding worthy of further exploration in larger and more diverse target cohorts. It is notable that the phenotypic variance captured by the AN PRS in global EDE scores in this study is equivalent to the phenotypic variance reported by Watson et al. (2019) for clinical AN cases. Phenotypic variance for feeding-related behaviors captured by the AN PRS was even greater than Watson et al. (2019). This may be attributable to the broader inclusion of individuals who may be classified unaffected in conventional case-control studies as they have not received a diagnosis of AN (or other ED), but who may sit very close to the boundary of that classification. This highlights the importance of investigating broader population-based phenotypes such as disordered eating, which includes individuals that would otherwise be misclassified as unaffected or potentially excluded from ED studies.

There are limited studies that have applied AN PRS as a predictor for specific disordered eating behaviors. Existing studies have investigated avoidance of eating, self-induced vomiting, and excessive exercise behaviors (Abdulkadir et al., 2022; Westwater et al., 2023), but the present study is the first to additionally include objective bulimic episodes as an outcome.

In the present study which investigated a solely female cohort, we identified a significant association between excessive exercise behavior and the AN PRS. Consistent with our results, Yilmaz et al. (2023) also reported a significant association between the AN PRS and excessive exercise. In contrast, Abdulkadir et al. (2022), using the same target cohort from the ALSPAC as Yilmaz et al. (2023), reported no significant relationship. With the same target sample and measures across the two studies, the difference in results is likely explained by sex differences. Abdulkadir et al. (2022) used a combined male and female ALSPAC cohort, whereas Yilmaz et al. (2023) investigated the predictive ability of the AN PRS in males and females from the ALSPAC cohort separately, with the AN PRS significantly predicting compulsive exercise in females, but not in males. This suggests that driven exercise may be associated with the AN PRS in females, but not in males. It is also important to highlight, however, that the AN GWAS from which the PRS is derived has predominantly female AN cases (Watson et al., 2019); this could impact the applicability of the PRS to cohorts that are male or that also include males.

AN PRS predicted avoidance of eating and self-induced vomiting behaviors in our study, in contrast to Abdulkadir et al. (2022), Yilmaz et al. (2023), and Westwater et al. (2023), who all reported no significant associations between AN PRS and these behaviors. The same GWAS summary statistics (Watson et al., 2019) were used to generate the AN PRSs as our study, however measures of disordered eating behaviors across the studies differed. Our study utilized a clinician-administered interview and assessed the lifetime presence of disordered eating behaviors, whereas Yilmaz et al. (2023) and Abdulkadir et al. (2022) used self-report measures of symptoms that occurred over the previous year only. Westwater et al. (2023) did use lifetime endorsement of disordered eating behaviors, however this was assessed through parent ratings using screener items, reducing reliability. These differences could account for our contrasting findings. The administration of a clinical interview – at three time points in adolescence – to the individuals themselves is likely to have improved our power to detect significant findings relative to the other studies, despite our much smaller target sample size. The discrepancies in our findings compared to Westwater et al. (2023) may also partially be explained by participant age differences. Participants were younger (age 9–11 years) in the study by Westwater et al. (2023), compared to the present study (age 12–19 years). Disordered eating behaviors typically emerge during adolescence, with bulimic-type behaviors such as self-induced vomiting often developing in early adulthood (Fairburn et al., 2003). Twin studies have also identified differences in genetic contributions to disordered eating across adolescence, with genetic influences increasing with age (Fairweather-Schmidt & Wade, 2015; Klump, Burt, McGue, & Iacono, 2007; Klump, McGue, & Iacono, 2000; O'Connor, Culbert, Mayhall, Burt, & Klump, 2020). This means the present study is likely to have captured both an increase in disordered eating behaviors and an increase in genetic contribution among participants, who were further along in adolescence compared to Westwater et al. (2023).

Limitations need to be considered when interpreting our results. Despite finding significant associations between the AN PRS and all disordered eating outcomes, our analyses were likely underpowered due to the sample size of the AN GWAS used to generate the PRS (Watson et al., 2019). The predictive power of a PRS is driven by the GWAS sample size, with larger samples increasing the power to detect robust associations (Hübel et al., 2018). This has been demonstrated in genomic studies on several psychiatric disorders, with the most notable being schizophrenia (Pantelis et al., 2014; Smoller et al., 2019; Wray et al., 2018). AN GWASs have only recently been sufficiently powered to detect significantly associated loci, and as AN GWAS sample sizes increase, the power to detect significant associations will also increase (Watson et al., 2021; Wray et al., 2014). Although PRSs utilize loci that fall below genome-wide significance, the power of PRSs significantly improves as the discovery cohort sample size increases.

Our target sample was relatively small, limited by the number of participants who provided blood samples for analyses. Replication in a larger adolescent female target cohort using the same global EDE and lifetime behavior outcome measures will enable more powerful analyses. A strength of the present study was use of a target sample from the general population, however this also meant there was a low number of participants who displayed disordered eating symptoms potentially reducing the power of our analyses. The mean global EDE score of our target sample was low (0.44 across all waves) and the number of participants endorsing lifetime behaviors was small, although prevalence of lifetime behaviors in our sample aligned with norms in comparable populations (Aardoom et al., 2012; Luce, Crowther, & Pole, 2008; Machado et al., 2014; Mond, Hay, Rodgers, & Owen, 2006). As previously mentioned the phenotypic variance in disordered eating captured by the AN PRS was equivalent to that reported in the most recent large GWAS (Watson et al., 2019).

Finally, it is important to highlight that the present study used a target sample of adolescent females of European ancestry, so findings cannot be generalized to other ages, genders, or ethnicities. This was further limited by the AN GWAS discovery data, which are currently restricted to individuals of European ancestry (Watson et al., 2019). ED research has primarily focused on females and is not representative of the diverse group of people who experience disordered eating (Huckins et al., 2022). Future studies need to utilize broader more diverse recruitment of people with EDs so findings can be more representative of those who experience symptoms.

Overall, the present study demonstrates that AN PRS predict disordered eating behaviors beyond those listed as AN diagnostic criterion, suggesting a shared genetic component across different disordered eating behaviors. This means the AN PRS may be useful in identifying individuals at greater risk of developing any disordered eating symptoms. It is important to also consider that the PRS may also be identifying shared eating anomalies and cognitive patterns common to both clinical and nonclinical eating disturbances, and may not be truly identifying prodromal states within the general population. It is critical to note that at this time, AN PRS does not have utility as a diagnostic or predictive tool, as presently it captures only a small part of the genetic contribution to our phenotypes. In time this variance captured will increase and it will be possible to establish if the AN PRS is a useful tool to identify those at risk of developing EDs. Incorporating PRSs into comprehensive risk assessment models that include clinical and psychological predictors may enhance risk assessment and intervention strategies. Future integration of PRSs with existing ED screening tools may have the advantage of identifying genetically susceptible individuals who may not have current disordered eating symptoms, allowing for targeted early interventions.

## Supporting information

Curtis et al. supplementary material Curtis et al. supplementary material

## Data Availability

All the data produced in the present study are available from the corresponding author upon reasonable request.

## Supplementary material

The supplementary material for this article can be found at https://doi.org/10.1017/S0033291724001727.
