# Supplementary material for: Anorexia nervosa polygenic risk, beyond diagnoses: relationship with adolescent disordered eating and behaviors in an Australian female twin population
Source: Psychol Med. 2024 Oct 23;54(13):3646–54. doi: 10.1017/S0033291724001727 (PMC11536114; doi:10.1017/S0033291724001727)
Supplement: Curtis et al. supplementary material [file S0033291724001727sup001.docx]

Supplementary Information

**Quality control, imputation, and polygenic risk score calculation**

DNA was extracted from whole blood and genotyped using the Infinium Global Screening Array V.1 (Illumina, CA) at Erasmus Medical Centre (Rotterdam). Raw genotypes were called using standard procedure in GenomeSudio as part of a large batch of 10,656 samples. These were exported on the Illumina ‘TOP’ strand, and checked for samples with low call rate (below 97%) and identifiable sex errors and IBD state with respect to the twins (no issues identified for these samples). Markers were filtered to remove a set of 560 markers which were duplicates or non-alignable to hg19, then those with GenTrain score < 0.6; missingness >5%; MAF < 1%; low Hardy-Weinberg p-value (<10-6) or failing sex-chromosome-specific filters (chrX: heterozygosity >1% for males), removing 189,039 markers; keeping 502,739. This batch was integrated with other identically quality-controlled batches from GSA chips, converted to the hg19 plus strand, and imputed for the 432,255 markers passing QC in all batches, using default settings and the HRCr1.1 reference on the Michigan Imputation Server (phased using Eagle; imputed using minimac4).

The PRSs for AN were calculated in PLINK version 1.9 (Chang et al., 2015) using GWAS summary statistics obtained from the Eating Disorders Working Group of the Psychiatric Genomics Consortium (PCG-ED). We used the Freeze 2 AN sample, which combined 33 cohorts to include data from 16,992 AN cases and 55,525 controls (Watson et al., 2019). Australian and New Zealander participants (N = 2,536 AN cases and 15,624 controls) were excluded from our PRS calculations to avoid potential crossover between base and target datasets. We performed clumping to account for linkage disequilibrium (LD) among SNPs and extract genetically independent variants. We retained the SNP with the smallest *p* value in each 1000kb window and removed those in LD with this SNP (*r^2^* > 0.1). PRS were calculated using the sum of all retained SNPs weighted by their effect sizes as estimated in the GWAS summary statistics (Watson et al., 2019). To optimise prediction accuracy and identify the *p* value threshold with the greatest prediction for each disordered eating outcome, PRS were calculated for eight different *p* value thresholds (*p* < 5 x 10^-8^, *p* < 1 x 10^-5^, *p* < 0.001, *p* < 0.01, *p* < 0.05, *p* < 0.1, *p* < 0.5, *p* < 1.0).

Chang, C. C., Chow, C. C., Tellier, L. C., Vattikuti, S., Purcell, S. M., & Lee, J. J. (2015). Second-generation PLINK: rising to the challenge of larger and richer datasets. *GigaScience, 4*(1), 7–7. <https://doi.org/10.1186/s13742-015-0047-8>

Watson, H. J., Yilmaz, Z., Thornton, L. M., Hübel, C., Coleman, J. R., Gaspar, H. A., . . . Mattheisen, M. (2019). Genome-wide association study identifies eight risk loci and implicates metabo-psychiatric origins for anorexia nervosa. *Nature Genetics, 51*(8), 1207–1214. <https://doi.org/10.1038/s41588-019-0439-2>

**Figure 3**

*
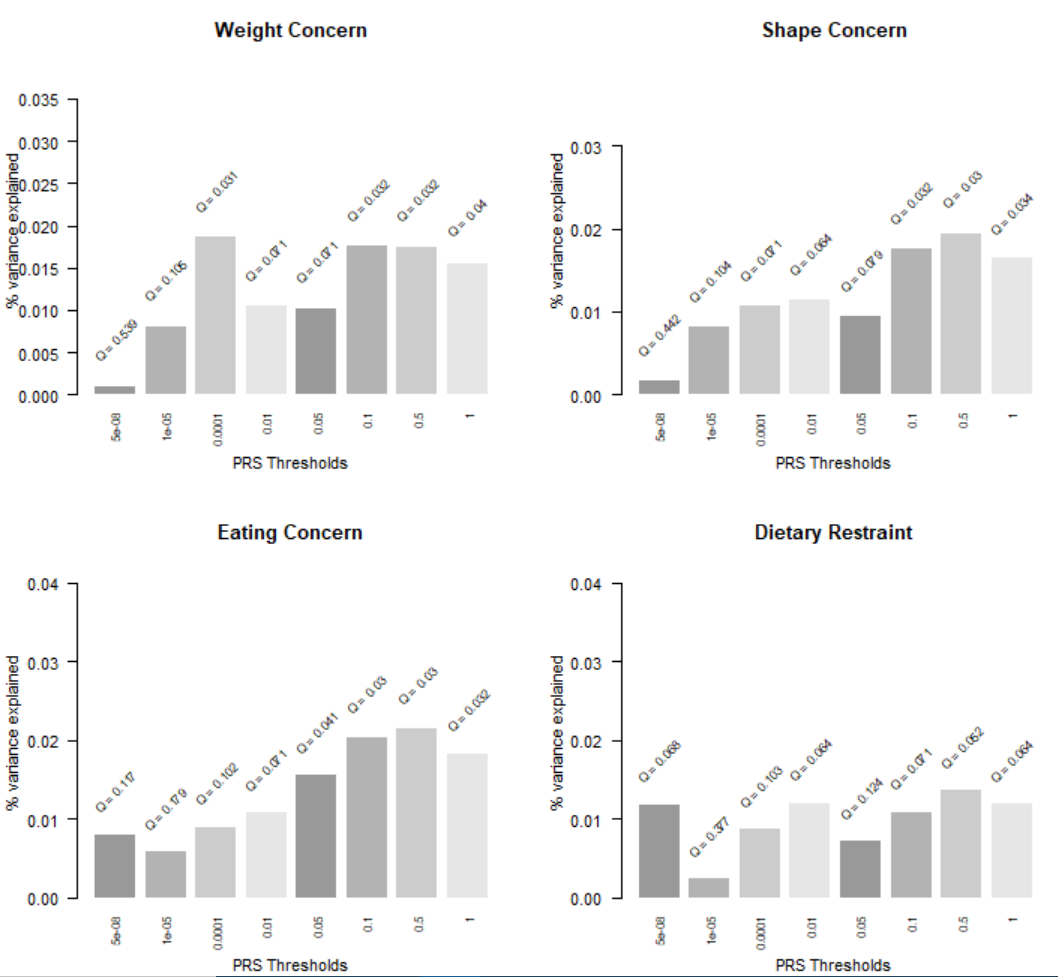
Associations between AN PRS and individual EDE subscales at eight p value thresholds.*
